# Tips and tricks for the persistent hiccup management in a Telemedicine encounter

**DOI:** 10.31744/einstein_journal/2022CE0155

**Published:** 2022-10-03

**Authors:** Tarso Augusto Duenhas Accorsi, Flavio Tocci Moreira, Karine De Amicis, Karen Francine Köhler, Eduardo Cordioli, Carlos Henrique Sartorato Pedrotti

**Affiliations:** 1 Hospital Israelita Albert Einstein São Paulo SP Brazil Hospital Israelita Albert Einstein, São Paulo, SP, Brazil.

Dear Editor,

The coronavirus disease 2019 (COVID-19) pandemic boosted Telemedicine encounters around the world, with physicians having to face a variety of clinical adversities daily. The conditions with a low level of scientific evidence to guide treatment, such as hiccups, are even more challenging in remote care. Hiccups (diaphragmatic and intercostal muscle contraction followed by laryngeal closure) are usually self-limited and benign after peripheral phrenic irritation. However, this symptom may be a life-threatening situation or related to severe diseases.^(1)^ Our Telemedicine center evaluated 245,633 patients from January 2020 to April 2021, and 39 (0.016%) patients had hiccups as the main complaint. Although possible, we did not observe frequent serious situations; only 2 (5.1%) patients were referred to the emergency department immediately. A specific step-by-step guideline is suggested, and some tips and tricks may help low-risk patients. First key point is the remote detection of red flags, which requires a very detailed evaluation. The classically defined persistent hiccups (lasting for more than 48 hours) increase the pre-test probability of severe underlying conditions, but this symptom is difficult to be assessed by Telemedicine, and referral is the only feasible option.^(2)^ We decided to consider intractable (more than 2 months) hiccups as mandatory face-to-face evaluation, as well as other temporal profiles related to neurological *deficits*, chest pain, abdominal pain, unexplained weight loss, ongoing malignancy or organic dysfunction.^(3)^ This evaluation may detect stroke, central nervous system infection, toxic-metabolic disturbances, cardiac ischemia, malignancy, and acute abdomen cases. Any observed symptom implies immediate emergency department referral.

After this filter, the second step is to identify obvious causes of phrenic stimulation, including overeating, ingestion of spicy foods, alcohol intake, carbonated beverages, very hot or very cold foods, swallowing of air while chewing gum or other causes of aerophagia, and excitement or emotional stress.^(4)^ The third step is a simple checklist of potential medicine-induced hiccups, such as anti-parkinsonism drugs, anesthetic agents, steroids and chemotherapeutical drugs.^(5)^ Psychogenic etiology is an exclusion criterion and should be considered, especially when there is an association with psychiatric symptoms or nighttime relief during sleep.^(6)^ Any triggers identified should be discontinued, and remote treatment may be considered the last step. However, there is insufficient evidence to guide the treatment of persistent or intractable hiccups with either pharmacological or non-pharmacological interventions. Therefore, treatment based on observations, even some anecdotal, but with no adverse effects, is recommended.

Before sending prescription medicine, a non-pharmacological approach may be tested during the remote encounter: 1) strategies to increase blood carbon dioxide and possibly decrease diaphragm contraction - breathe in a closed bag for 1 minute, and/or hold your breath for a few seconds;^(7)^ 2) gastric decompression and consequent reduction in phrenic stimulation, such as forced eructation; 3) induction of vagal reflex and regulation of phrenic stimulus - squatting, and/or drinking cold water;^(8)^ 4) deviation of nervous stimulus - sneeze with black pepper and/or ingestion of a spoon of sugar and/or grimace and forward tongue pull; 5) acupuncture evidence converted to *Do-in* - guide self-compression with the thumb at point BL2 (end of the right eyebrow, medially) for few seconds, few times. If no result, the pharmacotherapy includes chlorpromazine, gabapentin, baclofen, serotonergic agonists, prokinetics and lidocaine.^(9)^ Proton pump inhibitors can help in association with one of the other agents mentioned. Stewardship protocols for medications acting on the central nervous system are fundamental for a safe Telemedicine evaluation. Thus, metoclopramide is the cornerstone in this situation, and should be prescribed up to 24 hours after symptom relief.^(10)^
